# KLF2 Inhibits Chicken Preadipocyte Differentiation at Least in Part via Directly Repressing PPARγ Transcript Variant 1 Expression

**DOI:** 10.3389/fcell.2021.627102

**Published:** 2021-02-02

**Authors:** Tingting Cui, Jiaxin Huang, Yingning Sun, Bolin Ning, Fang Mu, Xin You, Yaqi Guo, Hui Li, Ning Wang

**Affiliations:** ^1^College of Animal Science and Technology, Northeast Agricultural University, Harbin, China; ^2^College of Life Science and Agriculture Forestry, Qiqihar University, Qiqihar, China; ^3^Key Laboratory of Chicken Genetics and Breeding, Ministry of Agriculture and Rural Affairs, Harbin, China; ^4^Key Laboratory of Animal Genetics, Breeding and Reproduction, Education Department of Heilongjiang Province, Harbin, China

**Keywords:** KLF2, PPARγ, promoter, chicken, preadipocyte differentiation

## Abstract

*Peroxisome proliferator-activated receptor gamma* (*PPAR*γ) is the master regulatory factor of preadipocyte differentiation. As a result of alternative splicing and alternative promoter usage, *PPAR*γ gene generates multiple transcript variants encoding two protein isoforms. Krüppel-like factor 2 (KLF2) plays a negative role in preadipocyte differentiation. However, its underlying mechanism remains incompletely understood. Here, we demonstrated that KLF2 inhibited the P1 promoter activity of the chicken *PPAR*γ gene. Bioinformatics analysis showed that the P1 promoter harbored a conserved putative KLF2 binding site, and mutation analysis showed that the KLF2 binding site was required for the KLF2-mediated transcription inhibition of the P1 promoter. ChIP, EMSA, and reporter gene assays showed that KLF2 could directly bind to the P1 promoter regardless of methylation status and reduced the P1 promoter activity. Consistently, histone modification analysis showed that H3K9me2 was enriched and H3K27ac was depleted in the P1 promoter upon KLF2 overexpression in ICP1 cells. Furthermore, gene expression analysis showed that KLF2 overexpression reduced the endogenous expression of *PPAR*γ *transcript variant 1* (*PPAR*γ*1*), which is driven by the P1 promoter, in DF1 and ICP1 cells, and that the inhibition of ICP1 cell differentiation by KLF2 overexpression was accompanied by the downregulation of *PPAR*γ*1* expression. Taken together, our results demonstrated that KLF2 inhibits chicken preadipocyte differentiation at least inpart via direct downregulation of *PPAR*γ*1* expression.

## Introduction

Adipose tissue is not only a depot for energy storage but is also the body's largest endocrine organ and regulates multiple physiological processes by secreting various endocrine and paracrine factors (Ràfols, 2013; Steiner and Lang, 2017; Trim et al., 2018). Adipose tissue expansion is caused by adipocyte hyperplasia and hypertrophy (Soukas et al., 2001). The adipocyte hyperplasia is controlled by the rate of preadipocyte proliferation, whereas the adipocyte hypertrophy is controlled by the degree of preadipocyte differentiation (Fujiwara et al., 2012; Laforest et al., 2015; Ghaben and Scherer, 2019). Preadipocyte differentiation involves a comprehensive network consisting of various transcription factors, such as peroxisome proliferator-activated receptor gamma (PPARγ) (Grimaldi, 2001; Elisabetta et al., 2002; Lee and Kai, 2014), CCAAT/enhancer-binding proteins (C/EBPs) (Tanaka et al., 2014), sterol regulatory element binding proteins (SREBPs) (Madsen et al., 2005), Sp1/Krüppel-like factors (Sp1/KLFs) (Rosen and MacDougald, 2006; Lee et al., 2019), and GATA binding proteins (GATAs) (Rosen and MacDougald, 2006; Lee et al., 2019). Among these transcription factors, PPARγ is considered the master regulator of adipogenesis (Elisabetta et al., 2002).

The Sp1/KLF family transcription factors have a conserved DNA-binding domain and three C2H2 zinc finger motifs in their C terminus (Banerjee et al., 2003; Wu and Wang, 2013). These molecules recognize and bind to GC-rich regions, including CACCC elements and GC box, via the conserved DNA-binding domain and positively or negatively regulate target gene expression in various biological processes (Banerjee et al., 2003; Wu and Wang, 2013). *In vivo* and *in vitro* studies have shown that many of the Sp1/KLF family members play critical roles in preadipocyte differentiation (Dan et al., 2005; Oishi et al., 2005; Enomoto et al., 2013; Soroush et al., 2013; Zhang et al., 2013; Kimura and Fujimori, 2014; Escalona-Nandez et al., 2015; Wang et al., 2015). For example, KLFs 4, 5, 6, 9, and 15 have been shown to promote preadipocyte differentiation (Dan et al., 2005; Enomoto et al., 2013; Mann et al., 2013; Soroush et al., 2013; Kimura and Fujimori, 2014; Escalona-Nandez et al., 2015), whereas KLFs 2, 3, 7, 10, and 16 inhibit preadipocyte differentiation (Banerjee et al., 2003; Zhang et al., 2013; Wang et al., 2015; Jang et al., 2016; Liu et al., 2018; Sun et al., 2020).

KLF2 is highly expressed in 3T3-L1 preadipocytes, and its expression decreases rapidly during preadipocyte differentiation (Banerjee et al., 2003). Moreover, KLF2 overexpression inhibited 3T3-L1 preadipocyte differentiation, as demonstrated by a reduction in intracellular lipid accumulation and gene expression of adipogenic marker genes *PPAR*γ, *C/EBP*α, and *ADD1/SREBP1* (Banerjee et al., 2003). Further studies have shown that KLF2 repressed 3T3-L1 preadipocyte differentiation by directly binding to the PPARγ2 promoter and inhibiting *PPAR*γ expression (Banerjee et al., 2003). Our previous study showed that chicken KLF2 overexpression suppressed chicken preadipocyte differentiation, which was similar to the results obtained for mouse KLF2 (Banerjee et al., 2003; Zhang et al., 2014). However, the underlying mechanism remains unclear.

Chicken *PPAR*γ gene possesses three alternative promoters designated P1, P2, and P3 and produces five different transcript variants (*PPAR*γ*s1–5*) and two protein isoforms (PPARγ1 and PPARγ2) (Duan et al., 2015). Among these five *PPAR*γ transcript variants, *PPAR*γ*1*, which is derived from the P1 promoter, was more highly expressed in chicken abdominal adipose tissues than in the other tested tissues (Duan et al., 2015). Besides, *PPAR*γ*1* was significantly highly expressed in abdominal adipose tissues of the fat chicken line compared with the lean chicken line from 2 to 7 weeks of age (Cui et al., 2018). Using bioinformatics analysis, we identified a putative Sp1/KLF binding site in the P1 promoter of chicken *PPAR*γ gene (Cui et al., 2018). These results led us to hypothesize that KLF2 inhibits chicken preadipocyte differentiation by directly regulating *PPAR*γ*1* expression. In this study, we demonstrated that KLF2 inhibits chicken preadipocyte differentiation at least partly by directly binding to and downregulating the P1 promoter of the chicken *PPAR*γ gene.

## Materials and Methods

### Cell Culture

The immortalized chicken preadipocyte cell line ICP1was generated by our lab (Wang et al., 2017). The chicken fibroblast cell line DF1 was purchased from the Institute of Biochemistry and Cell Biology, Chinese Academy of Sciences. ICP1 and DF1 cells were maintained in DMEM/F12 or DMEM (Gibco, USA) containing 10% fetal bovine serum (FBS) (Biological Industries, Germany), 0.1 mg/mL penicillin and 0.1 mg/mL streptomycin. The DF1 and ICP1 cells were cultured at 37°C and 5% CO_2_ in a humidified incubator.

### Plasmid Construction

The KLF2 expression plasmid (pCMV-Myc-KLF2) was previously constructed by our lab (Zhang et al., 2014). The lentivirus expressing KLF2 (LV-KLF2-Myc) and the lentivirus control (LV-Myc) were constructed and packaged by Hanbio (Shanghai, China). The P1 promoter reporter, and its series of reporters for 5′-truncation mutants and site-specific mutants of the P1 promoter were previously constructed in our lab (Cui et al., 2018). For preparation of the unmethylated and methylated P1 promoter reporter constructs, the plasmid pGL3P1-327/+108 was amplified in the *dam-/dcm- Escherichia coli* strain (TransGen Biotech, China). The P1 promoter fragment (−327 to +108 bp) was obtained by double digestion of the amplified pGL3P1-327/+108 with *Kpn*I and *Hin*dIII and was mock-treated or treated with M.SssI methylases (New England Biolabs, USA) to yield the unmethylated and methylated P1 promoter fragments (-327 to +108 bp) according to the manufacturer's instructions. Methylation was confirmed by restriction enzyme digestion using *Bst*UI (New England Biolabs, USA), which recognizes CGCG and is sensitive to methylation. The resulting unmethylated and methylated P1 promoter fragments (−327 to +108 bp) were religated into the pGL3-Basic vector to generate the unmethylated and methylated pGL3P1-327/+108.

### Transfection and Promoter Luciferase Reporter Assays

DF1 and ICP1 cells were cultured to 70–80% confluence and cotransfected with the indicated promoter reporter constructs along with pCMV-Myc or pCMV-Myc-KLF2 plus pRL-TK using Lipofectamine 2000 (Invitrogen, USA). The luciferase activities were determined at 48 h after cotransfection by a Dual-Luciferase Reporter System (Promega, USA).

### Western Blot Analysis

The DF1 or ICP1 cells were washed with cold PBS and lysed using RIPA Buffer containing 1% PMSF (Beyotime, China). Cellular proteins were separated by 12% SDS-PAGE and transferred onto nitrocellulose membranes (Millipore, USA). Then the membranes were blocked for 60 min and incubated overnight at 4°C with a Myc-specific antibody (1:1000, Beyotime, China), a PPARγ antibody (Cell Signaling Technology, USA) or a β-actin antibody (1:3000, Beyotime, China) as the primary antibody. After the blots were rinsed with PBST three times, they were incubated with HRP-conjugated anti-mouse (1:3000, Beyotime, China) for 60 min at room temperature as the secondary antibody. The blots were observed with an ECL Plus detection kit (Beyotime, China).

### Chromatin Immunoprecipitation Analysis

ChIP was conducted using a ChIP assay kit (Cell Signaling Technology, USA) as described previously (Cui et al., 2018). For the KLF2 ChIP assay, DF1 cells were transfected with pCMV-Myc-KLF2 or an empty pCMV-Myc vector. The transfected cells were fixed in 1% formaldehyde for 10 min and quenched in 0.125 M glycine for 5 min. Then, 100–900 bp DNA/protein fragments were obtained through micrococcal nuclease digestion. The samples were immunoprecipitated with Myc-specific antibody (Cell Signaling Technology, USA) and mouse IgG (Beyotime, China) as a control to analyse the binding of KLF2 to the P1 promoter. The purified DNA was amplified by qPCR with the ChIP-qPCR primer shown in Table 1. The input chromatin (2% nonimmunoprecipitated DNA) was used. The ChIP-qPCR data were calculated as previously detailed (Tatler et al., 2016; Cui et al., 2018).

**Table 1:** Primers or probes used in this study.

**Type**	**Primer name**	**Primer sequence**
mRNA qRT-PCR primer	*PPARγ1*	F: 5′-GGAGTTTATCCCACCAGAAG-3′
		R: 5′-AATCAACAGTGGTAAATGGC-3′
	*NONO*	F: 5′-AGAAGCAGCAGCAAGAAC-3′
		R: 5′-TCCTCCATCCTCCTCAGT-3′
	*FABP4*	F: 5′-ATGTGCGACCAGTTTGT-3′
		R: 5′-TCACCATTGATGCTGATAG-3′
	*G0S2*	F: 5′-CGGGGCGAAAGAGCTGAG-3′
		R: 5′-AGCACGTACAGCTTCACCAT-3′
	*GPDH*	F: 5′-ACCTCCCATCCCATACCGA-3′
		R: 5′-CCACTCCACGCTGCCAACA-3′
	*AdipoQ*	F: 5′-GCAACAACAACGGGGTCT-3′
		R: 5′-AGGGGAATTTTCTGGTACATAG-3′
ChIP qPCR primer	ChIP-qPCR P1	F: 5′-GAGCCCCGACCCGCGCAGCGCCCAC-3′
		R: 5′-ATAAACTCCCCGGGCCGGCCCATCC-3′
EMSA probes	CpG probe	F:5′-AGGCGGTGCCTGGCCGGTAGGATGGGCCGGCCCG-3′
		F:5′-CGGGCCGGCCCATCCTACCGGCCAGGCACCGCCT-3′
	CpG met4 probe	F:5′-AGG*C*GGTGCCTGGC*C*GGTAGGATGGGC*C*GGCC*C*G-3′
		F:5′-*C*GGGC*C*GGCCCATCCTAC*C*GGCCAGGCAC*C*GCCT-3′
	CpG met1 probe	F:5′-AGGCGGTGCCTGGC*C*GGTAGGATGGGCCGGCCCG-3′
		F:5′-CGGGCCGGCCCATCCTAC*C*GGCCAGGCACCGCCT-3′
	Cold probe	F:5′-AGGCGGTGCCTGGCCGGTAGGATGGGCCGGCCCG-3′
		F:5′-CGGGCCGGCCCATCCTACCGGCCAGGCACCGCCT-3′
	Cold mut probe	F:5′-AGTATTTGAATGGAATTTAGGATGGTAATGAAAT-3′
		F:5′-ATTTCATTACCATCCTAAATTCCATTCAAATACT-3′

For the histone ChIP assay, ICP1 cells were transfected with pCMV-Myc-KLF2 or an empty pCMV-Myc vector. After 48 h of transfection, the cells were fixed and quenched as described above. The samples were immunoprecipitated with the H3K9me2 antibody (Abcam, UK) and the H3K27ac antibody (ABclonal, China), and mouse IgG (Beyotime, China) and rabbit IgG (Cell Signaling Technology, USA) were used as controls for the H3K9me2 and H3K27ac antibodies, respectively. The rest of the ChIP procedure was the same as described above.

### Electrophoretic Mobility Shift Analysis

The KLF2 nuclear proteins were isolated from DF1 cells transfected with the pCMV-Myc-KLF2 construct using the nuclear extraction kit (Pierce, USA). The binding capacity of KLF2 to the DNA was detected by an EMSA Kit (Pierce, USA). Unmethylated and methylated biotin-labeled probes harboring the potential KLF2 binding site and the corresponding cold and mutant probes containing the P1 promoter sequence were synthesized by Genewiz (Beijing, China). The biotin-labeled unmethylated oligonucleotide probe was named the CpG probe (AGGCGGTGCCTGGCCGGTAGGATGGGCCGGCCCG, where the four CpG sites, which are underlined, were unmethylated), while the biotin-labeled methylated oligonucleotide probes were named the CpG met4 probe (AGG^m^CGGTGCCTGGC^m^CGGTAGGATGGGC^m^CGGCC^m^CG, where the four CpG sites that are underlined were fully methylated) and the CpG met1 probe (AGGCGGTGCCTGGC^m^CGGTAGGATGGGCCGGCCCG, where the 2nd CpG site, which is underlined, was methylated). The sequences of the sense and antisense probes are shown in Table 1. The labeled double-stranded probes (unmethylated and methylated) were incubated with nuclear extracts for 20 min. For competition assays, a 100- or 200-fold molar excess of unlabeled specific probes was added to the binding reactions before the labeled probes were added. For the supershift assay, 1 μg Myc-specific antibody (Cell Signaling Technology, USA) or normal mouse IgG as a negative control (Beyotime, China) was added to the reactions. The protein-DNA complexes were separated on a 6% polyacrylamide gel and observed with ECL reagent.

### Oil Red O Staining

The differentiated ICP1 cells were washed twice with cold PBS and then fixed with 4% paraformaldehyde for 30 min at 4°C as described previously (Zhang et al., 2019). Then, the cells were washed three times with cold PBS and twice with distilled water and stained with 1% freshly diluted oil red O for 15 min at room temperature in the dark. Finally, the cells were washed twice with PBS, and lipid droplets were imaged under a light microscope (Leica, USA). Finally, 100% isopropanol was added to the stained cells to extract the oil red O, and the absorbance was determined at 510 nm (Mu et al., 2020).

### RNA Isolation and Quantitative Real-Time RT-PCR

The total RNA was isolated from DF1 or ICP1 cells using TRIzol reagent (Invitrogen, USA), and the cDNA was generated with the ImProm-II^TM^ Reverse Transcription System (Promega, USA). RT-qPCR was performed using a SYBR Green PCR Master Mix (Roche, USA) on the 7500 Real-Time PCR System (Applied Biosystems, USA). The *NONO* gene was used as an internal control. The expression data were calculated using the 2^−ΔΔ*CT*^ relative quantification method. The primers used are shown in Table 1.

### Statistical Analysis

Data are expressed as the mean ± standard error of the mean (SEM). Comparisons between groups were performed using unpaired two-tailed Student's *t*-tests by Graph Pad Prism 5. The *p* values < 0.05 were considered significant (^*^p < 0.05, ^**^p < 0.01).

## Results

### KLF2 Negatively Regulates the Activity of the P1 Promoter

**Figure 1:**
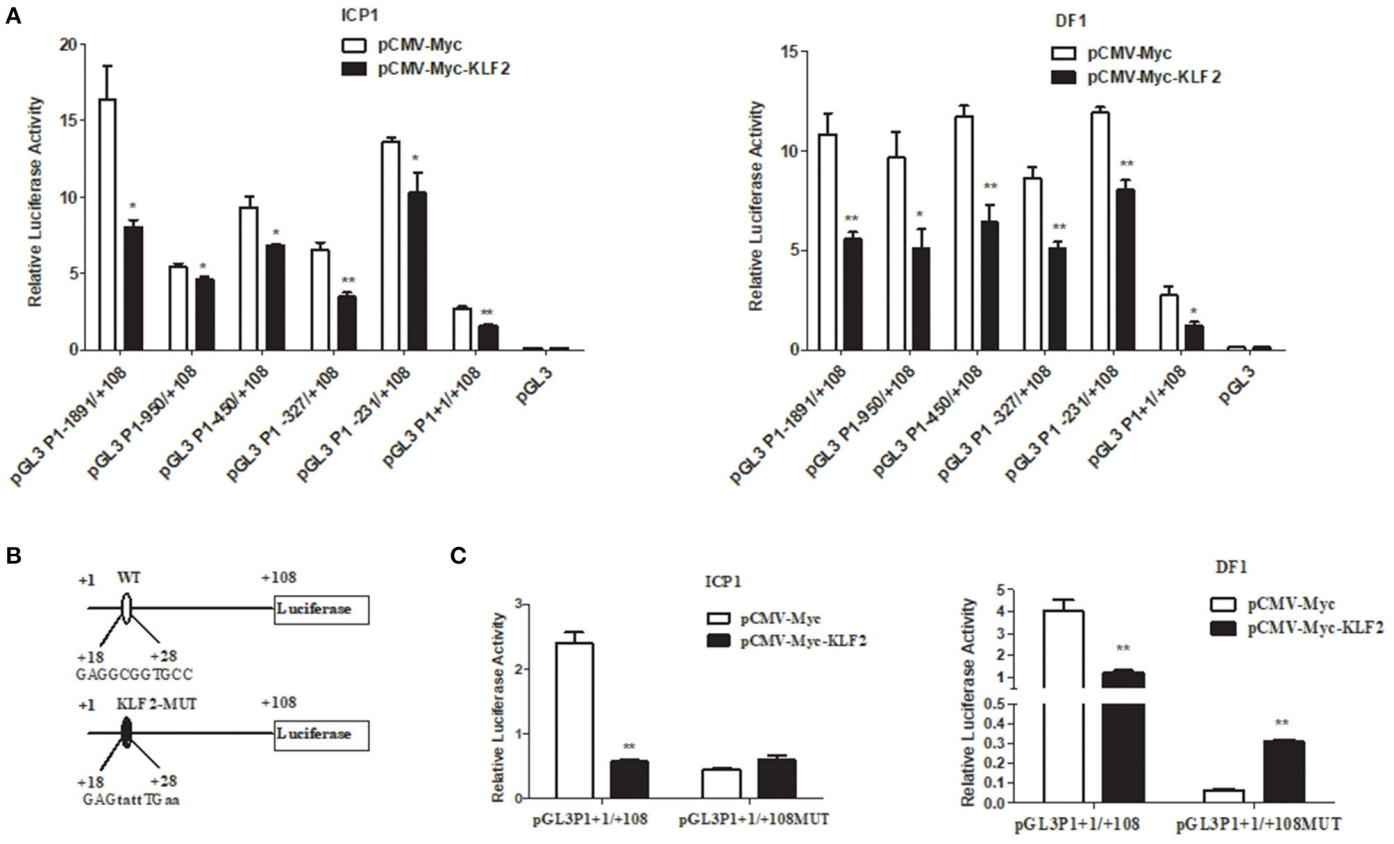
KLF2 represses the transcription of the chicken *PPAR*γ*1*. **(A)** Reporter assays showing the effects of KLF2 overexpression on P1 promoter activity in DF1 and ICP1 cells. Cells were cotransfected with the indicated 5′ deletion mutant reporters of the P1 promoter and pCMV-Myc-KLF2 or pCMV-Myc along with pRL-TK as an internal control. After 48 h of transfection, the luciferase activity was measured, and the data are presented as the mean ± SEM. Statistical significance was determined by a Student's *t*-test comparing cotransfection with the 5' deletion mutant reporters and pCMV-Myc-KLF2 vs. cotransfection with the 5′ deletion mutant reporters and the pCMV-Myc vector. *p < 0.05, **p < 0.01. **(B)** Schematic representation of the wild-type and KLF2 binding site mutants of the P1 promoter reporter constructs (pGL3P1+1/+108 and pGL3P1+1/+108MUT) used in the reporter gene assay. The nucleotide sequences of the wild-type and mutant KLF2 binding sites in the P1 promoter reporters (pGL3P1+1/+108 and pGL3P1+1/+108MUT). The mutation was generated by direct DNA synthesis and subsequent cloning into pGL3-basic, yielding pGL3P1+1/+108MUT. The KLF2 binding site is indicated by capital letters in pGL3P1+1/+108, and its mutated sequence is indicated by bold lowercase letters in pGL3P1+1/+108MUT. **(C)** The cells were cotransfected with pGL3P1+1/+108 and pGL3P1+1/+108MUT, along with pCMV-Myc-KLF2 or pCMV-Myc empty vector and pRL-TK as an internal control. The luciferase reporter gene assay was the same as described above in **(A)**. The data are presented as the mean ± SEM, and statistical analysis was performed by Student's *t*-test comparing the cotransfection of pGL3P1+1/+108 or pGL3P1+1/+108MUT with pCMV-Myc-KLF2 vs. cotransfection of pGL3P1+1/+108 or pGL3P1+1/+108MUT with the pCMV-Myc vector. *p < 0.05, **p < 0.01.

To determine whether the P1 promoter was regulated by KLF2, we cotransfected the P1 promoter construct (pGL3P1-1891/+108) or its series of 5′ deletion mutant constructs with the pCMV-Myc-KLF2 or empty pCMV-Myc vector plus the pRL-TK into DF1 or ICP1 cells. The reporter gene assay showed that compared to transfection of the empty vector pCMV-Myc, transfection with pCMV-Myc-KLF2 significantly repressed the luciferase reporter activity of the P1 promoter (pGL3P1-1891/+108) and its five 5' deletion mutants in both DF1 and ICP1 cells (*p* < 0.05 or *p* < 0.01) (Figure 1A). These data suggest that KLF2 negatively regulates P1 promoter activity and that the KLF2 binding site may be located in the +1 to +108 bp region of the P1 promoter.

Bioinformatics analysis of the +1 to +108 bp region using JASPAR showed that an Sp1/KLF binding site (GAGGCGGTGCC) was present at positions +18 to +28 bp. To demonstrate that the putative Sp1/KLF binding site is required for the KLF2-mediated transcription inhibition of the P1 promoter, we replaced this putative Sp1/KLF binding site with GAGTATTTGAA in the P1 promoter construct (pGL3P1+1/+108) (Figure 1B), and the resultant reporter was designated pGL3P1+1/+108MUT. We transfected pGL3P1+1/+108MUT with pCMV-Myc or pCMV-Myc-KLF2 plus the pRL-TK Renilla luciferase vector into DF1 and ICP1 cells. As expected, transfection of pCMV-Myc-KLF2 inhibited the promoter activity of pGL3P1+1/+108 compared with the transfection of pCMV-Myc (*p* < 0.01), but transfection of pCMV-Myc-KLF2 had no inhibitory effect on the promoter activity of pGL3P1+1/+108MUT (Figure 1C). Together, these results indicate that the GAGGCGGTGCC binding site is required for the KLF2-mediated transcription inhibition of the P1 promoter.

### KLF2 Directly Binds to the P1 Promoter

**Figure 2:**
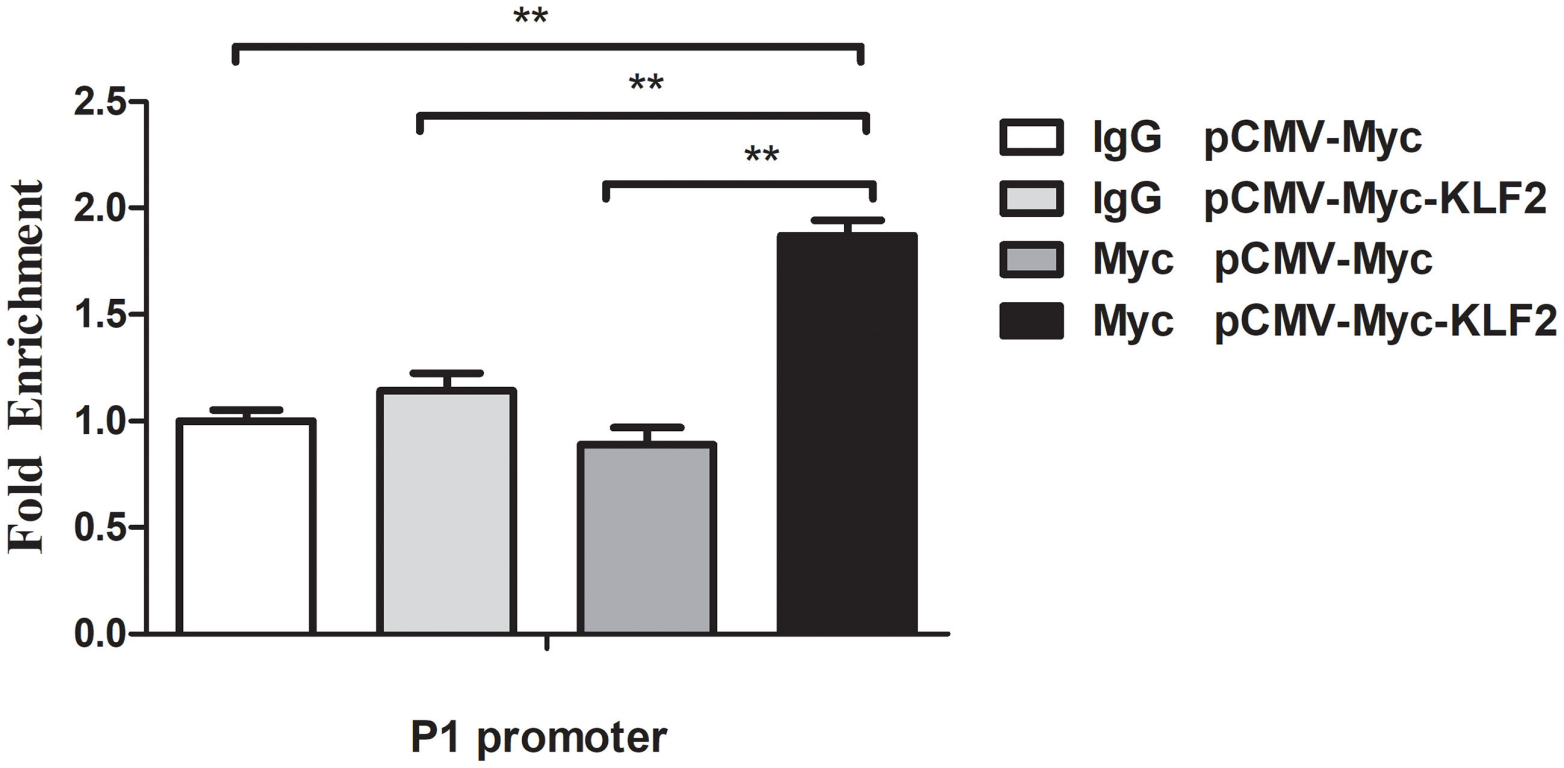
KLF2 directly binds to the P1 promoter. The binding of KLF2 to the P1 promoter was determined by ChIP-qPCR. DF1 cells were transfected with a pCMV-Myc-KLF2 or pCMV-Myc empty construct. At 48 h after transfection, ChIP-qPCR was performed using a Myc-specific antibody or mouse normal IgG. Nonimmunoprecipitated DNA (2%) was used as an input chromatin. Data were normalized to the negative control, which was provided by the cotransfection of DF1 cells with pCMV-Myc and immunoprecipitation with mouse IgG. All data represent the mean ±SEM. Statistical analysis was determined using Student's *t*-test, **p < 0.01.

A ChIP assay was performed to investigate whether KLF2 directly regulates the P1 promoter. pCMV-Myc or pCMV-Myc-KLF2 was transfected into DF1 cells, and a ChIP assay was performed with a Myc-specific antibody or normal mouse IgG as a negative control. qPCR was performed using a specific pair of primers(ChIP-qPCR P1)that covered the P1 promoter region (-60 to +52 bp) (Table 1). The results showed that the P1 promoter fragment was significantly enriched by 1.9-fold in the DNA immunoprecipitated by the Myc-specific antibody compared with the normal mouse IgG (*p* < 0.01) (Figure 2). In contrast, the P1 promoter fragment was not enriched in any of the other two negative controls, in which DF1 cells transfected with pCMV-Myc empty vector were immunoprecipitated by Myc-specific antibody or normal mouse IgG (*p* > 0.05). Collectively, these results suggest that KLF2 can directly bind to and regulate the P1 promoter (Figure 2).

### KLF2 Binds to the P1 Promoter Regardless of the DNA Methylation Status

**Figure 3:**
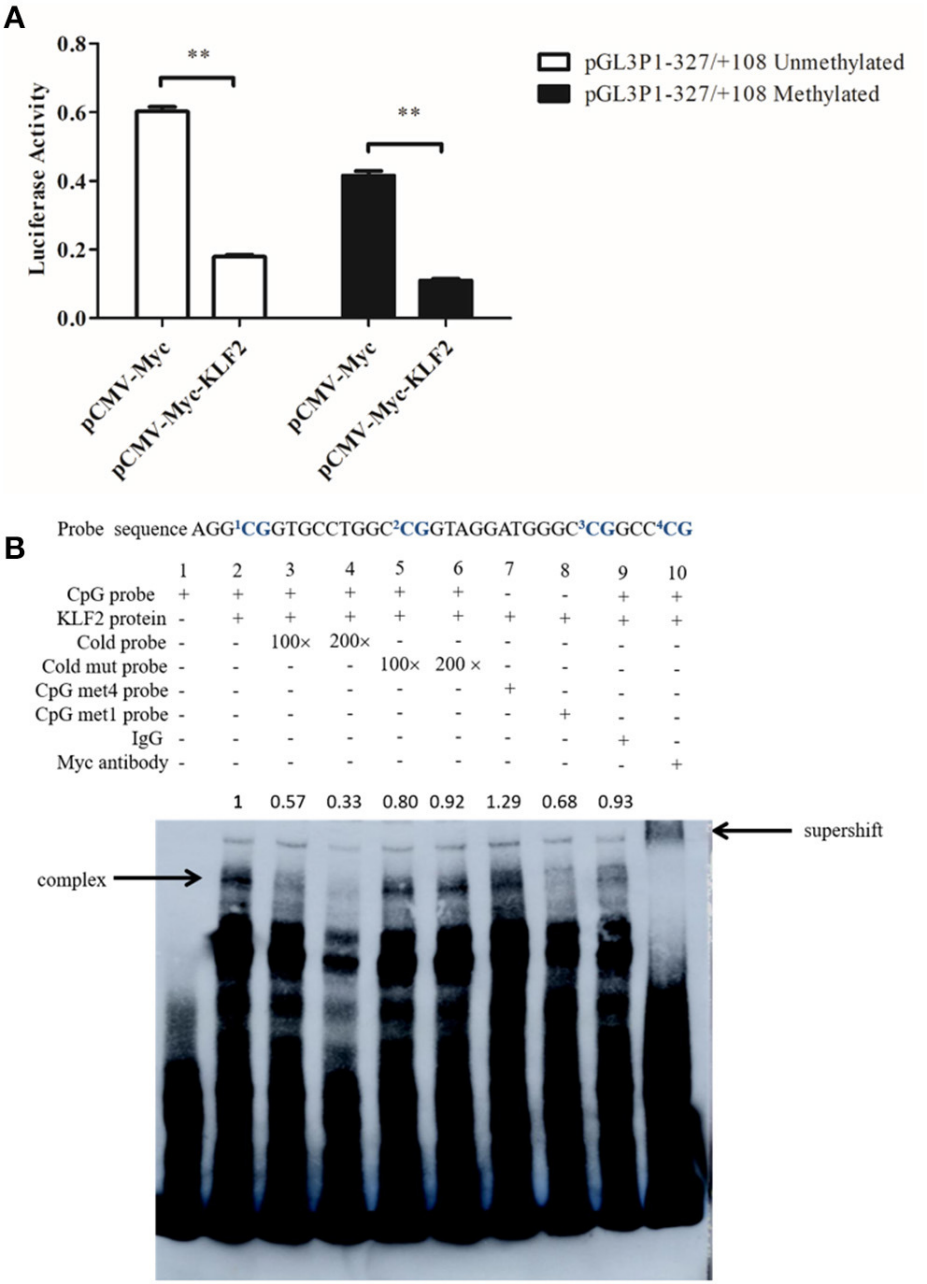
DNA methylation has no clear effect on KLF2-mediated suppression of the P1 promoter. **(A)** Effect of KLF2 overexpression on the activity of the unmethylated and methylated P1 promoters. DF1 cells were cotransfected with pCMV-Myc-KLF2 or pCMV-Myc empty vector, along with unmethylated or methylated P1 promoter reporter constructs and pRL-TK as an internal control. All data represent the mean ±SEM. Statistical analysis was determined by Student's *t*-test, **p < 0.01. **(B)** EMSA assay and supershift assays showing the binding of KLF2 to the unmethylated and methylated P1 promoters.

Our bioinformatics analysis revealed that the P1 promoter contained a CpG island at−333 to +52 bp and that the identified KLF2 binding site was located within this CpG island. This KLF2 binding site (+18 to +28 bp) contained a CpG site (underlined) (Figure 1B) designated CpG1, and three adjacent CpG sites (+35 to +54 bp) were designated CpGs 2–4. Our previous Sequenom MassArray analysis demonstrated that the methylation level of the P1 promoter CpG island in abdominal adipose tissue was significantly higher in the 7-week-old fat chickens than the lean chickens of the 19th generation of NEAUHLF (*p* < 0.05, data not shown). However, further pyrosequencing analysis showed that the methylation level of the CpG site located in the KLF2 binding site (CpG1, Figure 1B) was not significantly different between the fat and lean chicken lines. But the methylation levels of its three adjacent CpG sites (CpGs 2–4) tended to be higher in the fat lines than in the lean lines. Of this three adjacent CpGs, the CpG2 methylation level was significantly higher in the fat chicken lines than in the lean chicken lines (*p* < 0.05, data not shown). There are only 9 nucleotides between CpG1 and CpG2, whose methylation level was significantly higher in the fat chicken lines than in the lean chicken lines. These findings prompted us to test whether DNA methylation affects the KLF2-mediated regulation of the P1 promoter. To determine whether DNA methylation influences the KLF2-mediated suppression of P1 promoter activity, we performed a reporter assay. The unmethylated and methylated P1 promoter reporters (pGL3P1-321/+108) were individually transfected into DF1 cells with pCMV-Myc or pCMV-Myc-KLF2 plus pRL-TK. Notably, the results showed that KLF2 could efficiently inhibit the activities of both the unmethylated and methylated P1 promoters (*p* < 0.01) (Figure 3A). KLF2 tended to have a slightly higher inhibitory effect on the activity of the methylated P1 promoter than the unmethylated P1 promoter, but the difference was not statistically significant (*p* > 0.05).

Then, we further determined whether DNA methylation affects the binding of KLF2 to the P1 promoter by EMSA. The labeled P1 probe (+21 to +54 bp) (here after referred to as CpG probe), including CpGs 1–4, and two methylated counterpart probes (the CpG met4 probe, in which four CpGs were methylated, and the CpG met1 probe, in which CpG2 was methylated) were used in EMSAs. The EMSA results showed that a specific shifted band was observed with any of the three probes (the CpG probe, CpG met4 probe, and CpG met1 probe) (Figure 3B, lanes 2, 7, and 8), but no shifted band was observed with the nuclear protein extract from the empty vector-transfected cells (Figure 3B, lane 1). The addition of 100- or 200-fold unlabeled CpG probe (cold probe) eliminated KLF2 binding to the biotin-labeled probe (CpG probe), while the unlabeled mutant probe (cold mut probe) lost the ability to compete with the labeled probe (CpG probe) (Figure 3B, lanes 3–6). Additionally, a supershifted band was observed when the Myc-specific antibody, but not control IgG, was added (Figure 3B, lanes 9 and 10). Taken together, these data suggest that DNA methylation did not affect the KLF2-mediated suppression of the P1 promoter.

### KLF2 Overexpression Alters the Histone Modifications of the P1 Promoter

**Figure 4:**
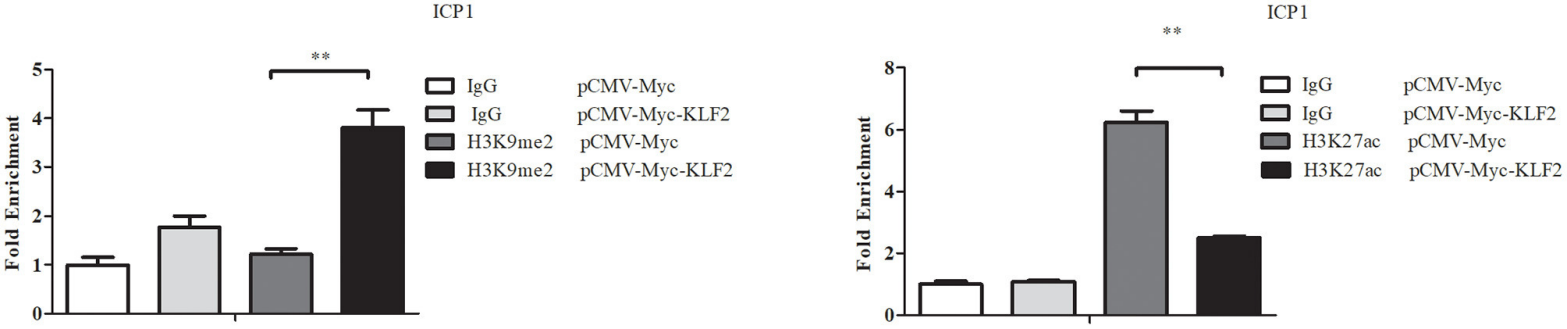
KLF2 overexpression alters the histone modifications H3K9me2 and H3K27ac in the P1 promoter. ICP1 cells were transfected with pCMV-Myc-KLF2 or pCMV-Myc empty vector. At 48 h after transfection, the cells were harvested and subjected to ChIP-qPCR to evaluate the enrichment of H3K9me2 and H3K27ac in the P1 promoter using H3K9me2 and H3K27ac antibodies, respectively. Mouse and rabbit normal IgG were used as a negative control. Nonimmunoprecipitated DNA (2%) was used as an input chromatin. Data were normalized to the negative control, which was provided by the cotransfection of cells with empty pCMV-Myc vector and immunoprecipitation with normal IgG. All data represent the mean ± SEM. Statistical significance was determined using Student's *t*-test. **p < 0.01.

A recent study showed that knockdown or overexpression of KLF2 increased or decreased histone marks (H3K9Ac and H4K8Ac) on the promoter of *Becn1* in RAW264.7 cells, a mouse monocytic cell line, resulting in up- or downregulation of Becn1 expression (Laha et al., 2019). We investigated whether KLF2 overexpression also alters histone modifications in the P1 promoter. Therefore, using ChIP-qPCR, we investigated the histone modifications (H3K9me2, a histone mark for gene inhibition, and H3K27ac, a histone mark for gene activation) of the P1 promoter in ICP1 cells, which were transfected with pCMV-Myc-KLF2 or pCMV-Myc. After 48 h of transfection, a ChIP assay was performed using mouse anti-H3K9me2 or normal control mouse IgG and rabbit anti-H3K27ac or normal control rabbit IgG. The enrichment of histone modifications in the P1 promoter was analyzed by qPCR with ChIP-qPCR P1 primers to amplify the P1 promoter region (Table 1). The results showed that H3K9me2 at the P1 promoter region was increased by 211.24% in the ICP1 cells transfected with pCMV-Myc-KLF2, compared with the cells transfected with pCMV-Myc (*p* < 0.01, Figure 4). However, H3K27ac at the P1 promoter region was decreased by 59.87% in the ICP1 cells transfected with pCMV-Myc-KLF2, compared with the cells transfected with pCMV-Myc (*p* < 0.01, Figure 4). These data are consistent with our finding that KLF2 negatively regulates the P1 promoter (Figure 1A).

### KLF2 Inhibits the Endogenous Expression of *PPARγ1*

**Figure 5:**
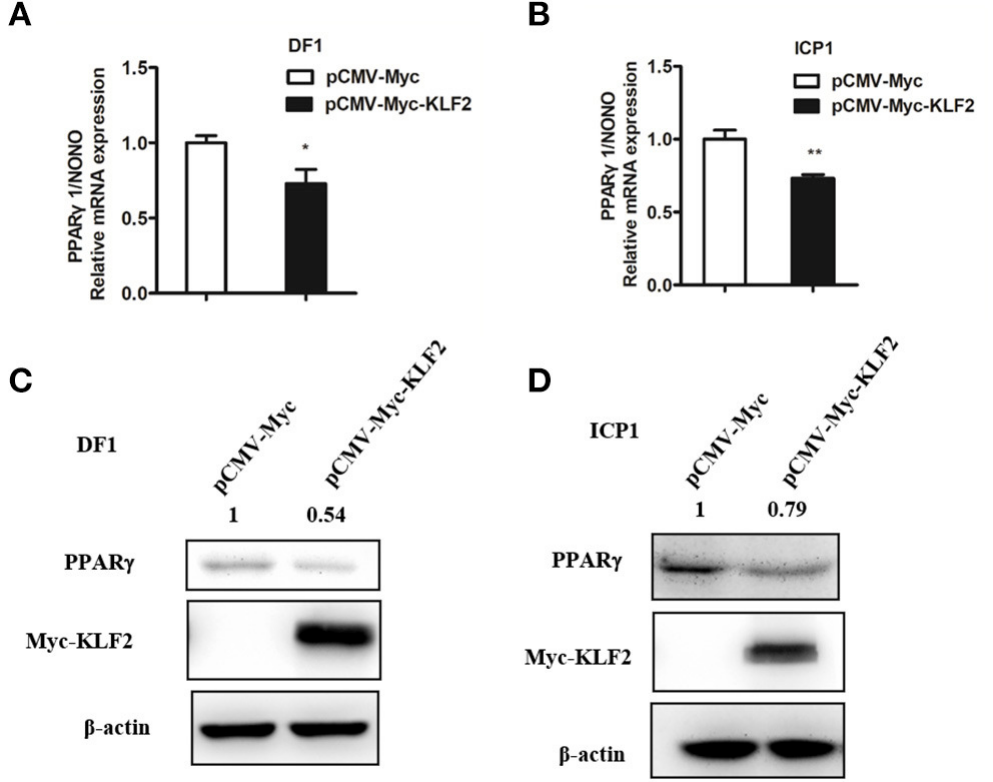
KLF2 overexpression inhibits the endogenous expression of PPARγ1. **(A,B)** qRT-PCR analysis showing the effect of KLF2 overexpression on endogenous PPARγ1 expression in DF1 and ICP1 cells. Cells were transfected with pCMV-Myc-KLF2 or pCMV-Myc empty vector. At 48 h after transfection, the relative expression level of *PPAR*γ*1* was detected by qRT-PCR. Chicken *NONO* was used as an internal control. **(C,D)** Western blot analysis showing the effect of KLF2 overexpression on the endogenous PPARγ protein expression in DF1 and ICP1 cells. The cells were transfected with either pCMV-Myc-KLF2 or pCMV-Myc empty vector. The cell lysates were harvested, and the expression of PPARγ, KLF2, and β-actin protein was detected by Western blotting using anti-PPARγ (PPARγ), anti-Myc (Myc), and anti-β-actin (β-actin). β-actin was used as a loading control. Quantification of the relative PPARγ protein levels (expressed as the percentage of the cells transfected with pCMV-Myc empty vector, set as 1.0) was performed by analyzing western blot data using the Image J software. Data are means ± SEM, *p < 0.05, **p < 0.01.

To further confirm that KLF2 negatively regulates the P1 promoter, we examined the effect of KLF2 overexpression on the endogenous expression of *PPAR*γ*1*, which is driven by the P1 promoter, in DF1 and ICP1 cells. pCMV-Myc-KLF2 or pCMV-Myc was transfected into DF1 and ICP1 cells, and after 48 h of transfection, the endogenous expression of *PPAR*γ*1* and the PPARγ protein level were determined using qRT-PCR and western blots. As expected, KLF2 protein was expressed in the pCMV-Myc-KLF2-transfected DF1 and ICP1 cells but not in the empty pCMV-Myc vector-transfected cells at 48 h after transfection. The qRT-PCR analysis showed that the endogenous *PPAR*γ*1* expression was decreased by 27.22 and 26.83%, respectively, in DF1 and ICP1 cells transfected with pCMV-Myc-KLF2, compared with the cells transfected with the empty pCMV-Myc (*p* < 0.05, Figures 5A,B). Consistently, western blot analysis showed that the endogenous PPARγ protein expression was decreased by 45.64 and 21.02%, respectively, in DF1 and ICP1 cells compared with the cells transfected with pCMV-Myc (Figures 5C,D). Taken together, these results demonstrated that KLF2 inhibits the endogenous expression of *PPAR*γ*1* in DF1 and ICP1 cells, indicating that KLF2 directly negatively regulates the P1 promoter (Figure 1).

### KLF2 Overexpression Inhibits Preadipocyte Differentiation at Least in Part via Downregulation of *PPARγ1*

**Figure 6:**
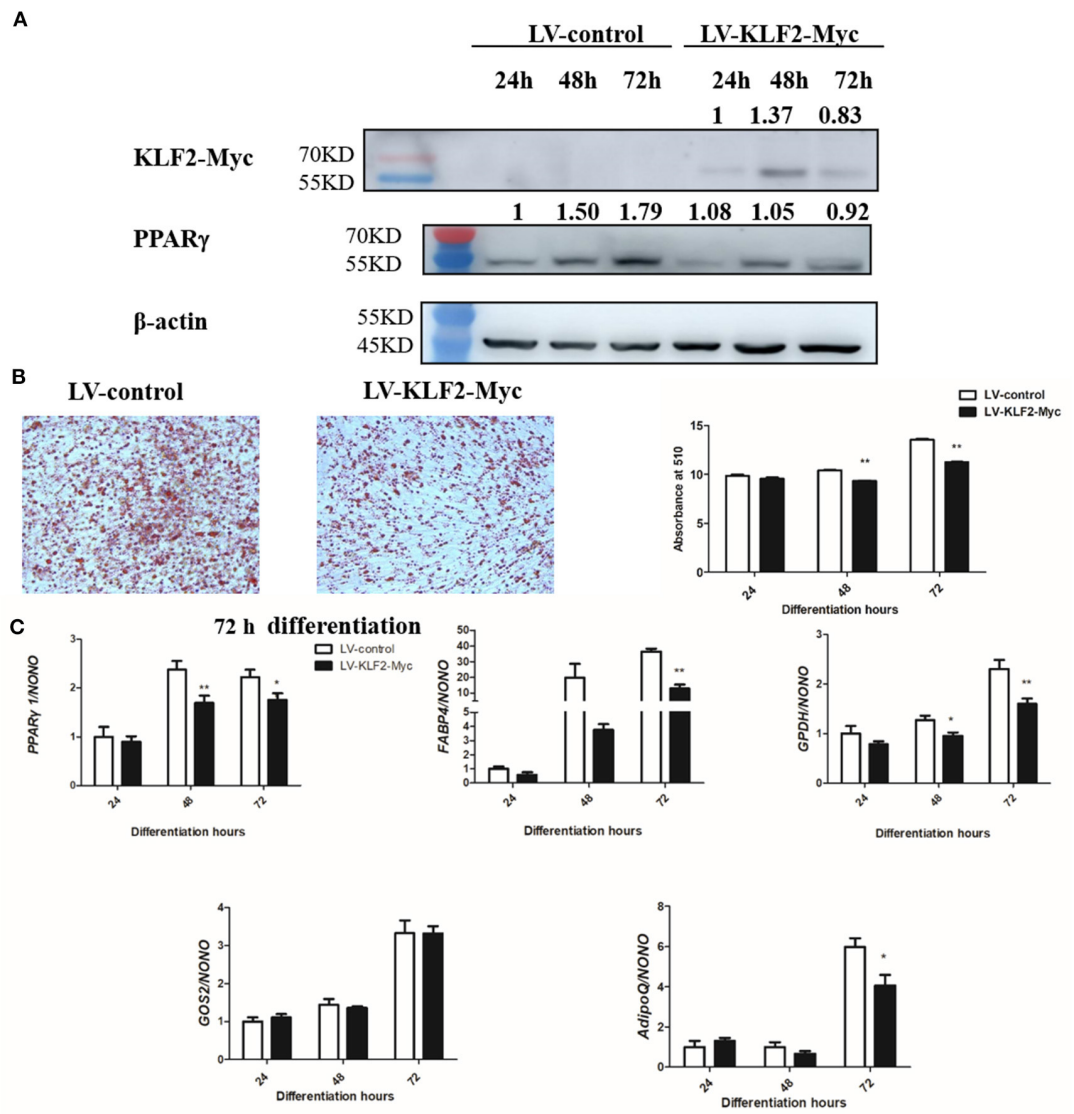
KLF2 overexpression inhibits the differentiation of ICP1 cells. **(A)** Western blot analysis of KLF2 and PPARγ protein expression during chicken preadipocyte differentiation. ICP1 cells were infected with lentivirus control (LV-Myc) or lentivirus expressing KLF2 (LV-KLF2-Myc), and then, 160 μM sodium oleate was added to induce the cells to differentiate. At the indicated time points, the cell lysates were harvested and immunoblotted with the indicated antibodies, respectively. **(B)** The effects of KLF2 overexpression on lipid droplet accumulation during ICP1 cell differentiation. Oil red O staining of the ICP1 cells infected with LV-Myc or LV-KLF2-Myc was performed at 72 h of differentiation (left two panels), and oil red O staining was quantified at 24, 48, and 72 h of differentiation (right panel). **(C)** The effects of KLF2 overexpression on adipogenic gene expression during the differentiation of ICP1 cells. The expression of adipogenic marker genes and *PPAR*γ*1* was determined at the indicated time points by quantitative real-time RT-PCR. All data represent the mean ± SEM. Statistical significance was determined using Student's *t*-test. *p < 0.05, **p < 0.01.

KLF2 overexpression was shown to inhibit chicken preadipocyte differentiation (Zhang et al., 2014). To investigate whether KLF2 inhibits chicken preadipocyte differentiation via downregulation of *PPAR*γ*1*, we investigated the effect of lentivirus-mediated KLF2 overexpression on the expression of *PPAR*γ*1* during chicken preadipocyte differentiation. ICP1 cells were infected with LV-KLF2-Myc or LV-Myc, and then, sodium oleate was used to induce the ICP1 cells to differentiate for 24, 48, and 72 h. Western blot analysis confirmed that KLF2 was successfully expressed in the cells infected with LV-KLF2-Myc at 24, 48, and 72 h of differentiation compared to the cells infected with LV-Myc (Figure 6A). As expected, KLF2 overexpression inhibited chicken preadipocyte differentiation as assayed by oil red O staining and quantitative real-time RT-PCR analysis of adipogenic marker genes. The lipid droplet accumulation in the cells infected with LV-KLF2-Myc was decreased by 10.43 and 17.10%, respectively, at 48 and 72 h of differentiation compared with the corresponding cells infected with LV-Myc (*p* < 0.01, Figure 6B).

In parallel, KLF2 overexpression reduced the expression levels of the adipogenic markers *GPDH* (48 and 72 h), *FABP4* (72 h), and *AdipoQ* (72 h) (*p* < 0.05 or *p* < 0.01, Figure 6C). *PPAR*γ*1* expression was reduced by 28.63 and 20.70%, respectively, in the cells infected with LV-KLF2-Myc at 48 and 72 h (*p* < 0.05, Figure 6C) compared with the corresponding cells infected with LV-Myc. Consistently, Western blot analysis showed that PPARγ protein expression was decreased by 30.00% and 48.60%, at 48 and 72 h of ICP1 cells differentiation, respectively compared with the corresponding cells infected with LV-Myc (Figure 6A). The suggesting that KLF2 inhibits chicken preadipocyte differentiation at least in part through downregulation of *PPAR*γ*1*.

## Discussion

Our previous study showed that *KLF2* mRNA expression was significantly decreased during the chicken preadipocyte differentiation (Zhang et al., 2014), but *PPAR*γ*1* expression continuously increased as this process proceeded (Cui et al., 2018). Further functional studies showed that the overexpression of KLF2 and PPARγ had opposite effects on chicken preadipocyte differentiation (Zhang et al., 2014). In the present study, we for the first time demonstrated that KLF2 inhibits chicken preadipocyte differentiation at least in part through downregulation of PPARγ1 expression.

In the present study, the promoter reporter gene assay, mutational analysis, EMSA, and ChIP-qPCR and gene expression analysis results supported that KLF2 negatively regulates chicken *PPAR*γ*1* expression by directly binding to the P1 promoter. Similar to our findings, it has been demonstrated that mouse KLF2 directly binds to and negatively regulates PPARγ2 promoter in 3T3-L1 preadipocytes (Banerjee et al., 2003). Consistently, sequence alignment showed that KLF2 binding site was conserved between chicken *PPAR*γ P1 promoter and mouse *PPAR*γ*2* promoter. Chicken PPARγ gene P1 promoter and mouse PPARγ2 promoter share some common features, for example, both of them contain KLF2, C/EBP, and AP1 binding sites, but also some substantial differences, mouse PPARγ2 promoter contains a TATA box but lack a CpG island (Zhu et al., 1995) whereas chicken P1 promoter contains a CpG island but lack a canonical TATA box.

In the present study, our results showed that transfection of pCMV-Myc-KLF2 significantly inhibited the luciferase reporter activity of the P1 promoter (pGL3P1+1/+108) (Figure 1C), but KLF2 binding site mutation completely abrogated the repression of KLF2 on pGL3P1+1/+108, suggesting that this binding site is required for KL2-mediated repression of the promoter activity of pGL3P1+1/+108. Unexpectedly, transfection of pCMV-Myc-KLF2 increased the luciferase activity of pGL3P1+1/+108MUT. This may be due to several reasons. First, the KLF2 binding site mutation may create a binding site for a positive transcription factor that is upregulated by KLF2 or activated by interaction with KLF2. Second, there are several transcription factor binding sites that overlap or close to this KLF2 binding site. These transcription factors may cooperatively form a complex to repress the P1 promoter, but in the presence of KLF2, the KLF2 binding site mutation may disrupt the inhibitory transcription complex, or result in change in conformation of the inhibitory transcription complex on the P1 promoter, leading to the increased luciferase activity. A similar phenomenon was also observed for Sp1, a member of Sp1/KLFs transcription factor family by (Roy et al., 2017). They found that the transfection of Sp1 expression vector significantly decreased luciferase activity of Atgl promoter, but when the Sp1 binding site was mutated. The Sp1 binding site mutation not only completely prevented the inhibitory effect of Sp1 on the Atgl promoter but also caused the increased Atgl promoter activity (Roy et al., 2017).

Several transcription factor binding sites overlap or near the vicinity of the KLF2 binding site on the P1 promoter (Cui et al., 2018), and these transcription factors may regulate transcription through competition for the same binding site. It has been shown that transcription factor Ventx1.1 competed with Xcad2 for binding to the same binding site in Ventx1.1 promoter to downregulate Ventx1.1 transcription (Kumar et al., 2019). To gain a better understanding of *PPAR*γ transcriptional regulation, it is worth investigating whether these transcription factors regulate the P1 promoter through competition in the future.

Our previous study showed chicken *PPAR*γ gene possesses three alternative promoters (P1, P2, and P3) (Duan et al., 2015). In the present study, we demonstrated that KLF2 directly binds to and negatively regulates the P1 promoter. Our previous reporter gene assay also showed that KLF2 inhibited the P3 promoter activity (Zhang et al., 2014). In addition, bioinformatics analysis showed that several putative KLF2 binding sites existed in the P2 promoter of chicken *PPAR*γ gene. These results suggest that during chicken preadipocyte differentiation, KLF2 may regulate these three promoters (P1, P2, and P3) of chicken *PPAR*γ gene simultaneously or sequentially.

Our present study showed that KLF2 repressed the P1 promoter activity and inhibited chicken preadipocyte differentiation. Similar to our findings, Banerjee et al. demonstrated that KLF2 remarkably reduced the activity of mouse *PPAR*γ*2* promoter and inhibited the 3T3-L1 differentiation (Banerjee et al., 2003). However, there are some difference between our and their results. Banerjee et al. demonstrated that mutation of KLF2 binding site alone was not sufficient to completely abrogated mouse KLF2-mediated inhibition of *PPAR*γ*2* promoter activity (Banerjee et al., 2003), but in our study, mutation of KLF2 binding site alone completely abrogated chicken KLF2-mediated inhibition of the P1 promoter activity. The discrepancy between our and their results could be due to species difference or the differences in experiment design (e.g., different size of promoter fragments and different cell lines used).

Promoter DNA methylation is negatively or positively correlated with gene expression (Adam and Farnham, 2013; Keren et al., 2014; Zhu et al., 2016; Gong et al., 2019). DNA methylation may alter the binding of transcription factors to their binding sites in the target promoters, resulting in the activation or repression of transcription (Zhu et al., 2016; Wang et al., 2017; Xu et al., 2018). For example, the transcription factors NRF1 and BmDeaf1 have been shown to bind to unmethylated or lowly methylated promoters but not to fully methylated or highly methylated promoters (Wang et al., 2017; Xu et al., 2018). Under some conditions, DNA methylation may enhance the binding of transcription factors, such as KLF4, C/EBPα, and C/EBPβ, to their methylated target promoters (Vikas et al., 2010; Mann et al., 2013; Zhu et al., 2016). A recent study identified KLF2 as a 5-methylcytosine (mC)-binding protein (Spruijt et al., 2013). In the present study, the reporter gene assay showed that DNA methylation had no clear effect on the KLF2-mediated inhibition of the P1 promoter activity (Figure 3A), and the EMSA demonstrated that KLF2 could directly bind to both the unmethylated and methylated P1 promoters (Figure 3B). Based on these results, we conclude that KLF2 regulates the P1 promoter regardless of the DNA methylation status.

**Figure 7:**
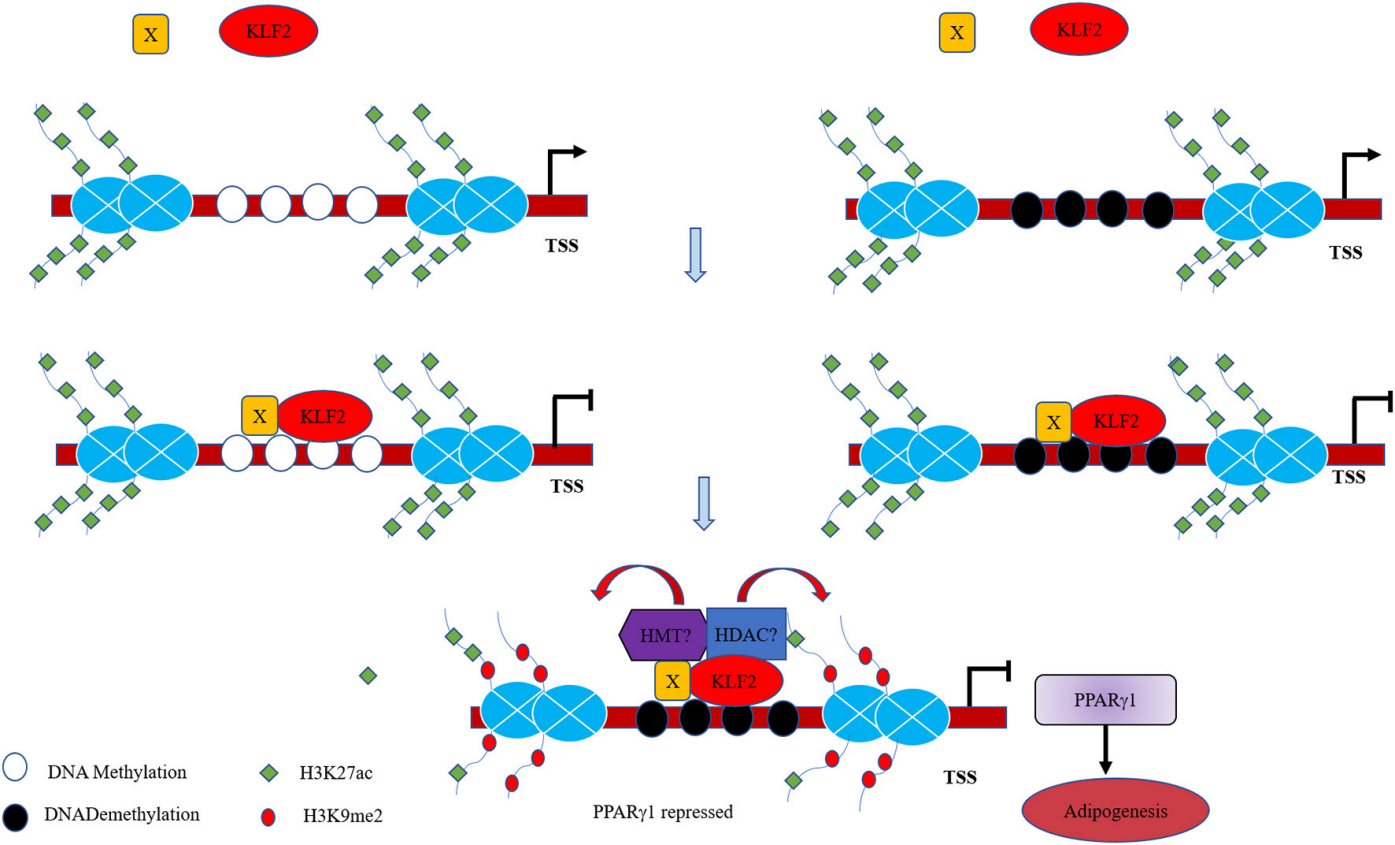
Proposed model of the mechanism for the transcriptional regulation of the P1 promoter and adipogenesis by KLF2. KLF2 directly binds to the unmethylated and methylated P1 promoter of the chicken *PPAR*γ gene with unknown cofactors (X), and recruit HMT and HDAC to the P1 promoter, which cause increased H3K9me2 and decreased H3K27ac, leading to inhibition of *PPAR*γ*1* expression and adipogenesis.

Transcription factors result in histone modifications in their target promoters by recruiting histone methylases/demethylases or histone acetylases/deacetylases (Liu et al., 2018; Guo et al., 2019). For example, KLF10 overexpression promoted the recruitment of HDAC1 to the C/EBPα promoter, leading to the depletion of acetylated histone H4 (acH4) in the C/EBPα promoter and inactivation of C/EBPα transcription (Liu et al., 2018). Recently, a study showed that in monocytes, KLF2 overexpression could decrease the enrichment of histone acetylation marks (H3K9Ac and H4K8Ac) in two promoter regions of *BECN1* (Laha et al., 2019). In this study, our results showed that KLF2 overexpression resulted in the enrichment of H3K9me2 and depletion of H3K27ac in the P1 promoter (Figure 4), consistent with our finding that KLF2 directly negatively regulates the P1 promoter. Based on our results, we hypothesize that KLF2 binds to the P1 promoter and recruits coregulators with diverse enzymatic activities, including histone deacetylases and histone methyltransferases, to the KLF2 binding site, these molecules modify histones on the P1 promoter, thereby promoting the formation of the chromatin conformation that inhibits the transcription of PPARγ1 and adipogenesis (Figure 7). In conclusion, we demonstrated that KLF2 directly binds to and inhibits the P1 promoter and that KLF2 inhibits chicken preadipocyte differentiation at least in part through downregulation of PPARγ1 expression.

## Data Availability Statement

The raw data supporting the conclusions of this article will be made available by the authors, without undue reservation.

## Author Contributions

TC performed the experiments, analyzed the data, and wrote the manuscript. NW conceived and supervised the study. NW and TC designed the experiments. JH and BN participated in the real-time PCR analysis. FM participated in western blot analysis. YS participated in the data analysis. All authors revised the manuscript.

## Conflict of Interest

The authors declare that the research was conducted in the absence of any commercial or financial relationships that could be construed as a potential conflict of interest.
